# Faces in the Crowd

**Published:** 1893-01-07

**Authors:** 


### MODERN LONDON.

### FACES IN THE CROWD.

ALTHOUGH we are constantly meeting persons we are not very desirous of interviewing, London is a very large place for a search after any one we are desirous of finding. And when successful in such a hunt, in nine cases out of ten we discover London life has effaced all interest in us from the mind of our friend of yore. Still disappointed Damon, if so "disposed," may yet obtain some amusement or even instruction from the London streets, especially when everyone has returned from Mont Blanc, or the Rockies, or Biarritz, or from wherever the Englishman and Englishwoman are wont to frequent to kill time, game, happiness, or misery; for, as some writer observed, "London is the university of the world: it attracts the rarest loveliness and the vilest ugliness, the ablest rogue and the most absolute fool." It not only attracts the healthy, but also those sick of any and every disease; for do not specialists abound in this modern Babylon of ours? Even yet, in remote country districts, an hazy idea of "Lunnon" streets paved with gold still floats in the yokel's mind, impelling not a few to seek their fortunes in the great metropolis, thus recruiting the population of this overgrown conglomeration of towns, without which addition sanitarians tell us the citizen race would die out.

Having failed to find our friend (possibly, if he knew, rather to his delight than otherwise), we take a walk along a principal thoroughfare, and at the risk of being somewhat jostled, or even run over if we attempt to cross, we fall into a moralising mood, being assisted towards this reckless frame of mind by the recollection that, if accident happens, the ambulance of the Hospitals Association will soon come to our assistance. Someone, however, rubs against us, and then we begin to wonder who he is amongst the countless numbers hurrying to and fro. A young man, fairly handsome and wholesome looking; light hair, freckled complexion and high cheek bones—evidently a young Scotchman come to seek his fortune in London, as so many of his countrymen have done before. He has the buoyant step of youth and health, and he turns up towards the Middlesex Hospital. He has several books and a roll of paper with him. Evidently a medical student, who for some reason or other prefers a London medical school to "walking the hospitals" in Edinburgh. Let us wish him success in his studies of what Byron designated the "destructive art of healing." And let us wish him good eye-sight, in these days of germs and microbes. For we are told in that too-learned book, "The Causation of Disease," that the smallest organic particle visible by the aid of the microscope contains nearly a million organic molecules, that a molecule of organic matter has some fifty elementary atoms, and that there are organic atoms as small in relation to the enveloping microscopical cell as the latter is to the earth on which we live!

While thinking of these things in, as our microscopical scientists will doubtless say, a ridiculous manner, we observe a young female—no doubt a servant girl sent out on an errand—and very probably one of the maid-of-all-work variety. She can scarcely be blamed if, having temporarily escaped from the basement and blackbeetles, she for a time solaces herself by an examination of various shop windows, and a fancy adornment of herself with the contents. It is simply astonishing the amount of work many of these general servant girls do, especially in lodging-houses, where they essay the impracticable feat of pleasing not only many masters, but what is still more impossible, of serving many mistresses also. But we fear our young friend looking in the shop windows will not much longer attempt impossibilities without a rest, for she walks lame, and as there is no appearance of disease judging from her face, we conclude she has "house-maid's" knee; and this affection is not to be cured without rest. This type of servant-girlism is quite a different one from that pervading private houses. Here we generally find sharp-witted London bred girls, who often look upon the household they enter as a caravanserai which they can leave at their pleasure, and who regard domestic service as the means by which they acquire the *summum bonum* of their present existence, which includes good living, fine dress, and evenings out.

But the passage of a man interrupts our thoughts. Undoubtedly a preacher, but whether a clergyman of the Church of England or not is questionable. On the whole, we are disposed to think not, for two reasons: first, because the white cravat is too dingy, and secondly because he is not accompanied by a wife and several children. Sydney Smith has been credited with saying, amongst other things which should not be credited to Sydney Smith, that we shall never find out in this world why shoemakers make our boots to pinch us. If he had said we shall never find out why so many curates have so many children, it would have been more to the point. But we are near the emporium of a second-hand bookseller, and we pause to scan the books and to survey the people also looking at the volumes. If any one would be an author, and is not deterred by what Solomon said, let him think awhile at an old book-stall. Here he may realise what "the bitter pangs of humbled genius" are, when the author sees his work, once fondly hoped might turn foolish thoughts into funds, exposed for sale at a few dirty coppers. But the book-stall is attractive to others than authors. This old gentleman, for instance, who scans the window with the air of a connoisseur, and who, wistfully turning an antique volume round and round, makes a halting step towards the shop door, and then hurriedly replaces the book and hobbles off. He did not intend stealing, and his poverty not his will consents to his going away without that coveted antique volume. It is a pity! For if we are not much mistaken that elderly gentleman is suffering under an early stage of locomotor ataxy, and the volume might have been a solace to him in the coming weeks when he will probably be confined to the most easy chair he can obtain. The young man standing next is of a different stamp. He simply remains to read, to glean information gratuitously, and has neither money for nor intention of purchasing. He finds it more pleasant to pursue an intellectual toil than to make entries in a ledger, or to wait behind a counter. Being out for the day, or half-day, he spends it at the book-stalls, where he wades unassisted through the dismal slough of learning as taught by the magistri of our youth—Roman emperors, early British manufactures, Cardinal Wolsey, Habeas Corpus, Napoleon Bonaparte, Romulus, Pitt, and all the rest of it. Many self-educated persons have chiefly acquired what they know by reading at book-stalls. Many persons have also caught that cold by standing at book-stalls which ended in the pneumonia sending them to that world where it is to be presumed books will not be required. The majority of people, however, pass book-stalls without taking any notice of them, for the school boards are now so absurdly imperative that everyone there educated begins to believe himself as wise as Sir Richard Temple.

Next we see a gentleman who has evidently strayed out of his proper sphere of Piccadilly, Pall Mall, or Bond-street. The glossy hat, the fashionable coat, the well-cut trousers, and the polished boots seem to stamp him as an *habitué* of the clubs, while the clean-shaved chin and the peculiar bearing stamps him as a military man. But he does not look happy! Perhaps he is one of the military unfortunates turned out of the army, by the absurd rules of the service, at forty years of age, just when he is most fit for his work, because he has not attained a certain rank in a certain number of years! The retired officer essaying to graze in this new pasture, generally ends by becoming a wine merchant's agent. Confound this street Arab coming whistling as shrilly as a steam engine, and as much out of tune as a trombone with a hole in it. Although he runs against us it is useless expostulating, for London street boys are as sharp for their age as the chick which begins to catch flies immediately it is out of the shell. By expostulation we should only become the butt of one whose idea of a joke is an impertinent observation and a loud guffaw. We really wish people would not write histories, touching and sweet, about London street children, because as a rule they are noisy and dirty. Better to give some money and leave them to Dr. Barnardo. But let us look at this young sailor rolling along. He is evidently of the now rare type of true British tar, who "loves the ocean breezes and loves the dashing spray." A pity we have not more of such men, for assuredly we shall want them, *when* our new men-of-war ships are built. Steamers have done much to diminish the number of real sailors. If sailors are required, they are scarcely to be found anywhere excepting in the North Sea fishing fleet. Closely following the young sailor comes a red-faced, wheezy, portly dame, with red flowers in her bonnet and red ribbons on her dress. Still, notwithstanding these decorations, there is a general air of seediness. The surmise may be wrong, but we cannot help thinking she keeps a lodging-house, would not disdain a glass of her lodger's whisky although preferring gin, likes single gentlemen as lodgers best, is a good hand at "hextras," and has a fatty heart.

But our notice of passers-by is interrupted by a crowd. Of course, it is a horse down. Now, although horses are very useful animals, they are often excessively stupid. They will sometimes shy the hundredth time they pass a stock or stone which they have seen ninety-nine times before without flinching. They are subject to unreasonable panics, when they run "a-muck" with as little consideration of consequences as a Malay drunk with bhang. They are always eating, and often lame. It is true they usually do not fall down if they can avoid doing so, but when they have subsided they do not always rise although able to do so. It is the case with the horse now down. He perversely refuses to get on his legs until he is well thrashed. Poor quadruped! He is certainly dazed, and perhaps half stunned. Means should be taken to prevent horses when down being promiscuously beaten. It may be required sometimes, but not always; and it is better to err on the side of mercy. We endeavour to explain to a burly policeman, who appears from somewhere round the corner, that when anyone is hurt by such an accident, there is always the Hospitals Association ambulance to be immediately obtained. But perhaps the burly policeman knows all about it, for he looks contemptuously and pityingly, and he replies: "Now, move on here! move on here!" So, having every respect for constituted authority —since Bumble was abolished—we move on.

